# Aging Intensifies Myeloperoxidase Activity after Ischemic Stroke

**DOI:** 10.14336/AD.2023.1640

**Published:** 2024-08-30

**Authors:** Negin Jalali Motlagh, Cuihua Wang, Hyung-Hwan Kim, Yonghyun Jun, Daeki Kim, Seeun Lee, John W. Chen

**Affiliations:** ^1^Institute for Innovation in Imaging, Department of Radiology, Massachusetts General Hospital, Harvard Medical School, Charlestown, MA 02114, USA.; ^2^Center for Systems Biology, Massachusetts General Hospital, Harvard Medical School, Charlestown, MA 02114, USA.; ^3^Stroke and Neurovascular Regulation Laboratory, Massachusetts General Hospital, Harvard Medical School, Charlestown, MA 02129, USA.

**Keywords:** Aging, stroke, neuroinflammation, myeloperoxidase

## Abstract

Abnormally elevated oxidative stress underlies many diseases and contributes to aging. The myeloid enzyme myeloperoxidase (MPO) generates oxidative stress and contributes to damage after stroke. How aging changes MPO in stroke has not been studied. We aimed to determine the effects aging has on MPO and how these changes contribute to age-related differences in outcomes after ischemic stroke. To investigate tissue MPO activity we developed MPO Activatable Fluorescent Agent (MAFA). We found that aged mice exhibited worse neurological outcomes and higher mortality within the first few days after stroke. Accordingly, neuronal loss was higher in aged mice on day 3. MAFA imaging revealed that aged brains have markedly higher MPO activity that increased after stroke on day 3 compared to young adult brains. Correspondingly, we found more Iba1^+^ cells in aged brains compared to young adult brains before and after stroke. Interestingly, we found decreased percentage of MPO^+^ cells and lower MPO protein levels in aged on day 3, suggesting that most Iba1^+^ cells in aged mice have degranulated and secreted MPO in response to stroke. By day 10 MPO activity and Iba1^+^ cells decreased in both age groups, although MPO activity remained higher in aged mice. MPO inhibition in aged mice decreased MAFA signal and Iba1^+^ cells and improved neurobehavioral outcomes to near young adult stroke mice levels and improved mortality rate. While aging is an unmodifiable risk, by uncovering the connection between aging and MPO-related changes after stroke, new therapies can be developed to mitigate these adverse changes brought upon by aging.

## INTRODUCTION

Stroke is currently the fourth-leading cause of death and the primary cause of long-term disability worldwide [1]. Its incidence increases by a factor of 100 between the age of 40 and 80 [2, 3], and its severity also increases with age [4]. Ischemic stroke is the most common stroke type, accounting for > 80 % of all strokes [5]. While age is a known key risk factor for stroke, it remains unclear how aging increases the risk and severity of stroke. It has been postulated that aging can affect the immune response [6-8]. Such altered immune response has been observed after ischemia in older age groups including humans [9, 10] as well as rodents [11-14], and have been found to increase the risk of post-stroke dementia [15].

One of the key products of an abnormal immune response is elevated oxidative stress, which can result in the accumulation of oxidative damage to lipids, DNA, and proteins by reactive oxygen/nitrogen species that over time may contribute to aging [16, 17]. On the other hand, oxidative stress also plays roles in homeostasis and cell signaling [18], and can sometimes be protective [19]. It is when oxidative species are uncontrollably elevated and overwhelm the antioxidant balance that excess oxidants can contribute to aging and disease. However, studies assessing oxidative stress in aging have been hampered by the lack of reliable and specific tools to detect and locate elevated oxidative stress and oxidant production. Thus, a tool that can reliably and specifically report on the location of excessive oxidant production with high sensitivity is needed to allow the identification of aberrant oxidative stress in normal and abnormal aging and provide a deeper understanding of how aging affects oxidative stress to alter outcomes in ischemic stroke and other age-related diseases.

One of the key producers of oxidative stress is myeloperoxidase (MPO) [20, 21]. After ischemic stroke, due to the destruction of the blood-brain barrier, immune cells such as neutrophils and macrophages infiltrate and microglia are activated in the ischemic areas [22] and release MPO [23, 24]. MPO is a highly oxidative enzyme, capable of generating both oxidative and nitrosative stress. After activation with H_2_O_2_, MPO oxidizes substrates (e.g., chloride (Cl̄), bromide (Br̄), nitrite (NO_2_¯), tyrosine (Tyr)) to potent oxidants (hypochlorous acid (HOCl) or hypobromous acid (HOBr)) and free radicals (nitrogen dioxide (^?^NO_2_) or tyrosyl radical (Tyr^?^)). These free radicals and oxidants are more potent than O_2_^-?^ and H_2_O_2_ for oxidizing biomolecules and inducing cellular injury [25, 26]. Thus, elevated MPO activity is indicative of increased oxidant production and the presence of excessive oxidative stress.

MPO activity in the ischemic cortex is elevated as early as 6 h after ischemia onset, peaked at day 3, and gradually returned to near basal level by the third week in transient middle cerebral artery occlusion (tMCAO) and permanent MCAO (pMCAO) animal models [23, 27]. Interestingly, tMCAO had a significantly higher MPO level in the ischemic cortex than the pMCAO model after stroke onset, indicating that perfusion increases MPO and associated oxidative stress [27, 28]. As such, inhibiting MPO activity or congenital absence of MPO can improve neuroprotection [29] and neurogenesis [29] to decrease infarct size, improve functional outcomes, and lower mortality [30] after experimental ischemic stroke. However, the effect aging has on MPO in ischemic stroke has not been explored. In this study, we aimed to determine the effects aging has on MPO and how these changes contribute to age-related differences in outcomes after ischemic stroke. To clearly identify the changes in tissue MPO activity, we developed a new fluorescent imaging probe for MPO activity called MAFA to image tissue MPO activity in the ischemic brain.

## MATERIALS AND METHODS

### Animal model of focal cerebral ischemia

Animal experiments were approved by the local institutional animal care. All mice were maintained in a federally approved animal facility at our institution and allowed to acclimate for one week prior to the start of the experiments. Transient focal cerebral ischemia was induced in 60 male and female aged C57BL/6J mice (10-14 months old) and 46 male and female young adult C57BL/6J mice (2-3 months old). To investigate the effects MPO inhibition has on aged mice after stroke, 14 mice from the aged group were treated intraperitoneally twice daily with 80 mg/kg of 4-aminobenzoic acid hydrazide (ABAH), a specific and irreversible MPO inhibitor [31].

Mice were anesthetized with 2% isoflurane. A rectal temperature probe was inserted to monitor and maintain a constant animal core temperature of 37 ± 0.5 °C using a temperature controller (TC-1000, CWE INC, Ardmore, PA). was induced by MCAO as described by Li et al [32]. Briefly, an 8-0 nylon monofilament suture (ETHICON LLC, Puerto Rico, USA) coated with silicone rubber (Heraeus Kulzer LLC, South Bend, IN) and hardener was inserted into the left internal carotid artery and advanced approximately 10 mm distal to the carotid bifurcation to occlude the origin of the middle cerebral artery. The thread was carefully withdrawn 30 min after MCAO to induce I/R injury. On day 3 and day 10 after surgery, blood was collected for flow cytometry and brains were collected for flow cytometry, ELISA, and immunofluorescent staining. To investigate MAFA specificity, focal cerebral ischemia was induced in three C57BL/6J male and female aged mice (10-14 months old, Jackson Laboratories) and three aged-matched (10-14 months old), MPO knockout mice (B6.129X1-*Mpo^tm1Lus^*/J, Jackson Laboratories). Fresh frozen brains were collected on day 3 after induction for immunofluorescent staining. Animals with intracranial hemorrhages were excluded from the study (n = 5 from aged n = 2 from young adults and n = 2 from aged ABAH-treated). because hemorrhages can exaggerate the inflammatory and immune response in ischemic areas [33].

### Behavior tests

We performed the following three types of behavior tests with eleven male and female aged C57BL/6J mice (10-14 months old) and six male and female young adult C57BL/6J mice (2-3 months old).

*Neurological deficit score:* Neurologic deficit score (NDS) was scored into four categories (modified from Clark and colleagues) daily for up to 10 days after MCAO stroke induction (Supplementary Fig. 4) [34].

*Hanging wire test:* Mice were placed on a metal wire (60 cm long) suspended 48 cm above a foam pad.; the latency to when the animal falls within a 60-sec period is recorded every day after MCAO.

*Cylinder test:* Mice were placed in a glass cylinder (10 cm diameter and 15 cm height). The number of times the animal used each forelimb or both forelimbs to contact the wall of the cylinder within 10 minutes were counted for each animal daily for 10 days after MCAO and a percentage relative to the total number of contacts was computed.

### Flow cytometry

Mice were transcardially perfused with 20-30 mL ice-cold PBS. The ischemic hemisphere of brains was collected and stored on ice in PBS. Then the brains were mechanically disrupted with a glass homogenizer and passed through a 40 µm nylon cell strainer (BD Biosciences, San Jose, CA), and single cells were isolated from 30/70 Percoll (GE Healthcare, Boston, MA) gradient, centrifuged for 5 minutes at 350 G and resuspended in FACS buffer (PBS with 0.5% BSA) for surface antibody staining. Antibody panels used were as described below. Briefly, cells were first stained with anti-CD16/CD32 (Bio Legend, San Diego, CA, 1:200) to block Fc binding sites for 20 minutes, washed with FACS buffer 3 times and then stained with anti-mouse antibodies against anti-NK1.1-PE/Cy7 (Bio Legend); anti-B220/CD45-PE/Cy7 (Bio Legend); anti-CD3-PE/Cy7 (Invitrogen); anti-CD11b-PE (Bio Legend); anti-Ly6G-FITC (Bio Legend); anti-CD45.2-PB (Bio Legend); anti-MPO-Biotin (Hycult). For intracellular staining, cells were fixed and permeabilized using Cytofix/Cytoperm (BD Bioscience) after staining for cell surface markers. Streptavidin-conjugated Brilliant Violet 605 secondary antibody (Bio Legend) was used to label biotinylated anti-MPO. For MPO-positive cells analysis in blood, blood was lysed in red blood cell lysis buffer, and staining was performed in a similar setup as that for brain staining once cells were in a single-cell suspension. On flow cytometry, CD11b^+^CD45^high^cells represented peripherally derived CNS-infiltrating M**φ**/activated Mg while CD11b^+^CD45 ^intermediate/low^ represented resident Mg [35]. These cells were then further identified as MPO^+^ and MPO^-^ cells. Data were acquired with a flow cytometer (Fortessa X-20; BD Biosciences and Aurora) and analyzed with BD FlowJo software (version 10.4). The representative flow scheme is shown in Supplementary Fig. 5.

### Enzyme-linked immunosorbent assay

Ischemic brain hemispheres were homogenized separately in 500 µL of cetyltrimethylammonium buffer (50 mM potassium phosphate at pH 6.0 with 50 mM CTAB) by a tissue homogenizer (Fisher Scientific). The samples were then sonicated for 30 seconds and centrifuged at 5668 G for 15 minutes. The supernatant was used for protein analysis with a BCA protein assay kit (Thermo Scientific). The supernatant was then used for MPO (R&D system, Cat# dy3667) and beta-3 tubulin (MyBioSource, Cat# mbs9398627), a neuronal integrity marker [36] ELISA following manufacture instructions.

**Figure 1.**
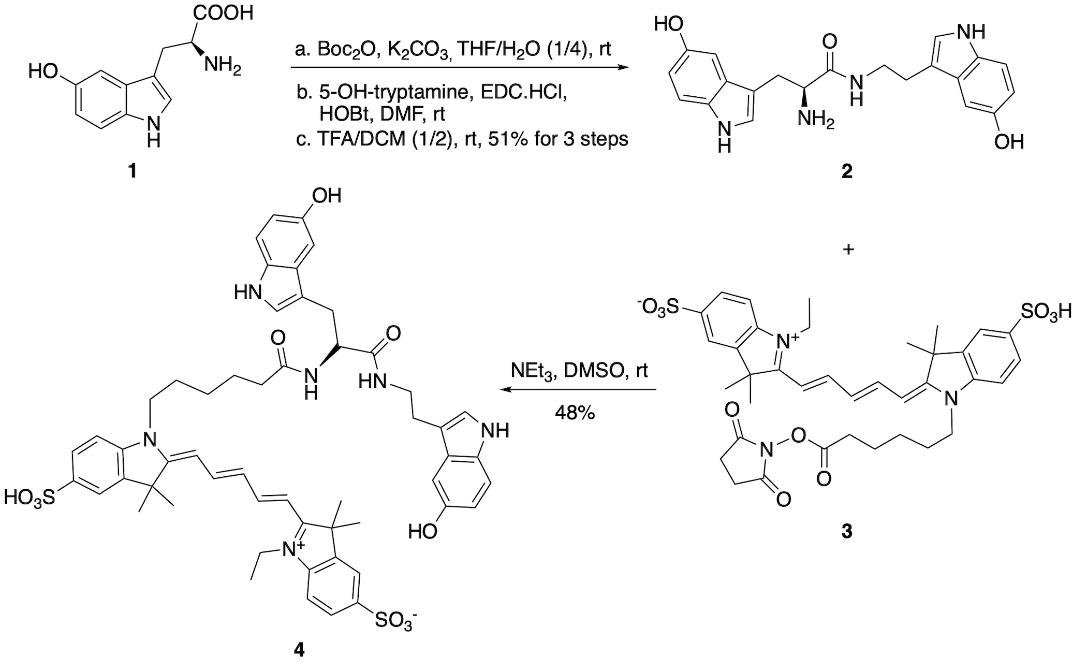
Chemical synthesis of MPO Activatable Fluorescent Agent (MAFA).

### Synthesis of MPO Activatable Fluorescent Agent (MAFA, Fig. 1)

Starting from 5-hydroxytryptaphan (compound 1), the intermediate 2 was obtained in three steps in a total yield of 51% as described previously [37]. Then a solution of intermediate 2 (8.4 mg, 1.2 equiv.) in dimethyl sulfoxide (DMSO 1 mL) was added triethylamine (6 uL) and stirred for 20 min at room temperature. A solution of cy5-NHS ester (compound 3) in DMSO (0.5 mL) was added to the above solution and stirred for another 1 h at the same temperature. The reaction solution underwent high-performance liquid chromatography to give the desired compound 4 (MAFA) in a yield of 48%. High-resolution mass spectrometry (HRMS) of MAFA: found 1017.3888 (Calc. 1017.3885) as shown in (Fig. 1 and Supplementary Fig. 6).

### Immunofluorescent staining

Fresh-frozen brains were cut in serial cross sections (8-10 µm thick). The sections were fixed with 4% PFA for 2-3 minutes at room temperature (RT). The slides were incubated in blocking buffer (5% Donkey serum (EMD Millipore Cat# 3915503) and 0.3% triton x-100 (Millipore Cat# t8787-50 ml in PBS) at RT for 1 h, followed by incubating in blocking buffer containing the primary antibody overnight at 4?. Primary antibodies were rabbit anti-MPO (1:300, Thermo Fisher, Cat#PA5-16672), rat anti-NeuN (1:500, Abcam Cat# ab279297), rat anti-Iba1(1:300, Abcam Cat# ab 283346) and for MAFA validation, Rabbit anti-chlorotyrosine (1:500, Hycult, Cat# HP5002). On the second day, slides were rinsed with PBS and then incubated with secondary antibodies at RT for 1 h. Secondary antibodies were anti-rabbit Alexa flour 555 (1:300, Invitrogen Cat# xf347096) and anti-rat Dy Light 488 (1:300, Invitrogen, Cat# xg3652581). The sections were washed 3 times with PBS following incubating with MAFA (1:300 of stock solution 10 mM in DMSO and 1 mM of 3% H_2_O_2_) for 30 minutes at RT. The sections were mounted in an antifade mounting medium (Vectashield, Cat# zj0808) and DAPI. Images were captured with the Nikon DS-Ri2 model microscope connected to Prime BSI Express.

**Figure 2.**
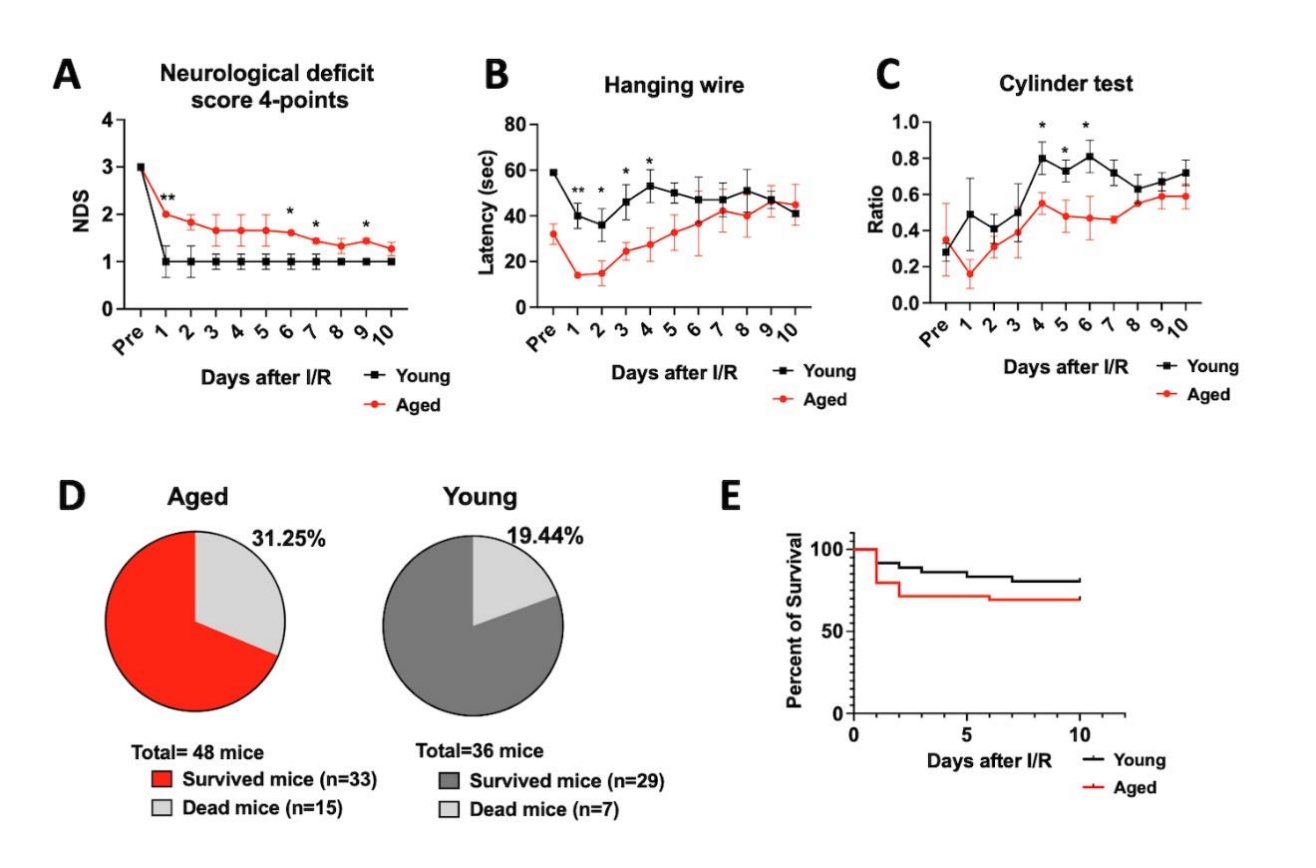
**Aging worsens neurological deficits and behavior outcomes in ischemic stroke. (A)** Neurological deficits scores were significantly higher in aged mice compared to young adults after stroke (n = 11 for the aged and n = 6 for young adults for all behavior tests and neurological deficits scores). **(B)** Forelimb grip strength was measured by the hanging wire test and the latency to fall was quantified. **(C)** The ratio of using the contralateral limb was measured with cylinder tests. Significant differences were found in both hanging wire and cylinder tests between aged and young adults after stroke. **(D)** The mortality rate was higher in (log-rank) curve of aged and young adult mice after stroke. Statistical values were determined by unpaired t-tests.

### Statistical analysis

Results were reported as mean ± standard error of mean (SEM). Neurological deficit scores and behavior tests between the aged and young adult groups were analyzed using unpaired two-tailed t-test. Log-rank (Mantel-Cox test) was applied to compare the mortality rate between the aged untreated and aged ABAH-treated group. Differences between the two groups for flow cytometry, ELISA, and immunostaining were assessed by the Mann-Whitney test. Three to four regions of interest within the infarct area were chosen for each mouse to calculate the integrated density of immunofluorescent images using ImageJ (v 2.1.0). We used GraphPad Prism (v.9.5.1, GraphPad Software) for statistical analysis. *P* < 0.05 was considered statistically significant.

**Figure 3.**
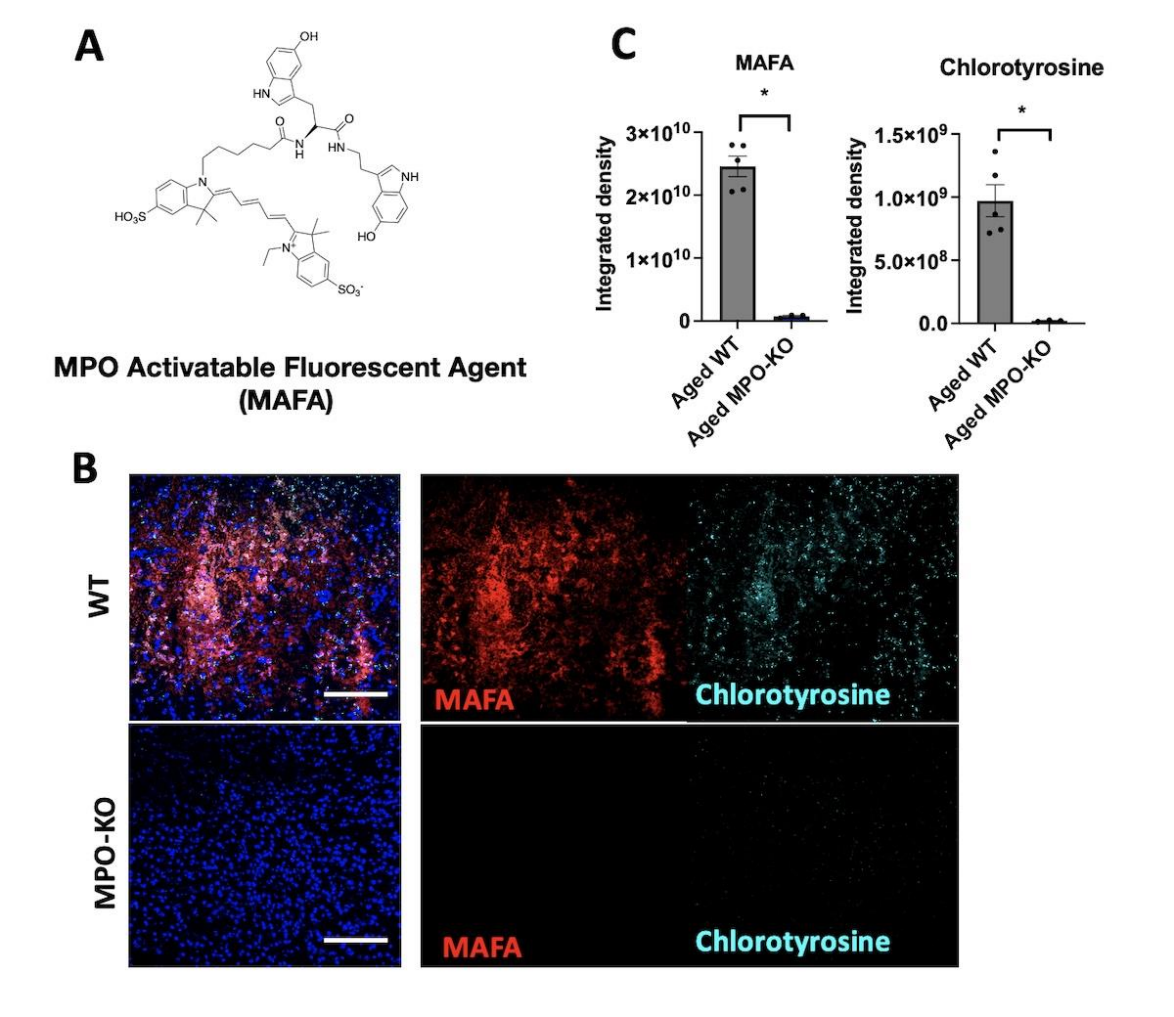
**Structure and validation of MAFA. (A)** Structure of MPO Activatable Fluorescent Agent (MAFA). **(B, C)** Immunofluorescent images of fresh frozen brains. Imaging of MPO activity with MAFA revealed a significantly higher fluorescent signal in wild-type as compared to MPO-KO mice on day 3 after stroke (n = 5 wild-type mice, n = 3 MPO-KO mice, the scale bar = 100 µm). Correspondingly, chlorotyrosine expression was remarkedly higher in the wild type as compared to MPO-KO mice (n = 5 wild-type mice, n = 3 MPO-KO mice, Mann-Whitney test) the scale bar = 100 µm). *, *p* <0.05.

## RESULTS

### Aged mice exhibit significantly worse neurological deficits scores and behavior outcomes compared to young adult mice

To assess neurological outcomes after stroke in both aged and young adult mice, the neurological deficit scores were recorded daily after stroke induction. Both young adults and aged mice showed clear neurological deficits on the 1^st^ day after surgery. However, young adult mice showed rapid improvement in their deficits post-stroke whereas aged mice exhibited a slower recovery rate and continued to show functional deficits (Fig. 2A). Additional tests evaluating motor coordination similarly revealed a larger motor deficit in the aged mice compared to the young adult mice: in the hanging wire test young adult mice could hold on to a wire longer compared to aged mice after stroke, and in the cylinder test aged mice showed more asymmetric limb preference compared to young mice (Fig. 2B and 2C). These findings revealed that functional damage was exacerbated in aged mice compared to young mice after stroke. Aged mice not only had significantly worse functional outcomes compared to young mice but also had a higher stroke-induced mortality rate and lower survival rate than young adult mice after stroke (Fig. 2D and 2E). Notably, mortality was highest in the aged group within the first three days after stroke induction.

**Figure 4.**
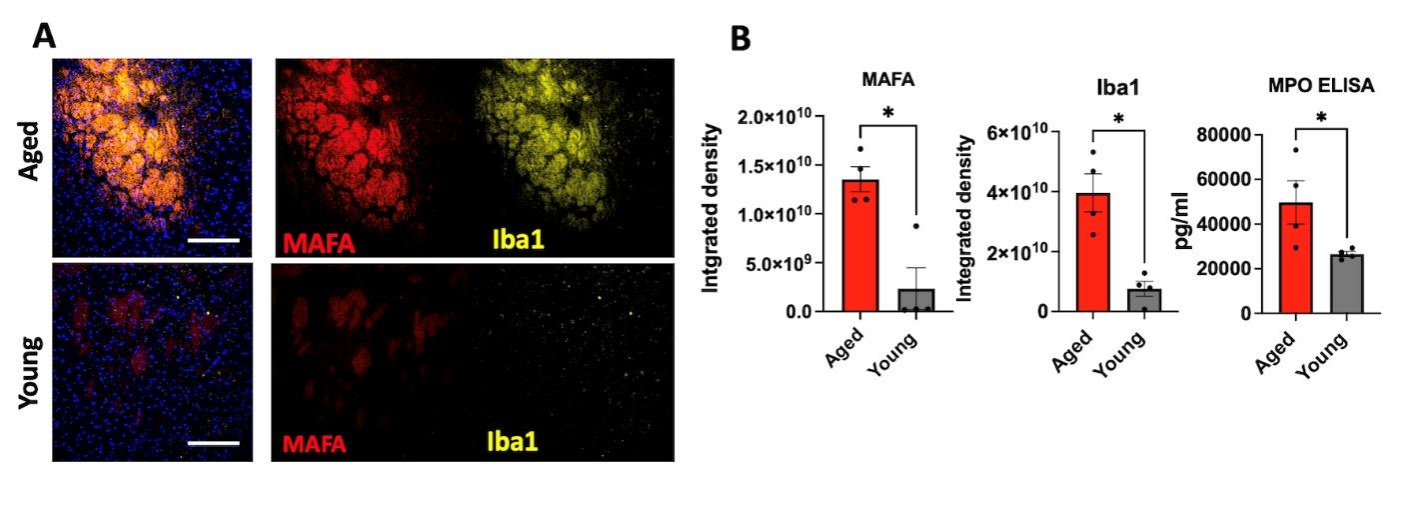
**MAFA signals increased with aging. (A)** Representative immunofluorescent images illustrating MAFA and Iba1 signal in the thalamus region of aged and young adult brains prior to stroke (n = 4 mice per group, the scale bar = 100 µm). **(B)** Quantification showed that MAFA and Iba1 signals were significantly higher in aged brains as compared to those of young adults. MPO ELISA also showed higher amount of MPO protein in the aged brains compared to that in the young adult brains (*, *p* < 0.05, Mann-Whitney test).

### Validation of MAFA tissue imaging in ischemic brain tissues

MAFA was designed as an activatable fluorescent probe for the detection of MPO activity (Fig. 3). As a substrate for MPO, MPO oxidizes MAFA leading to the formation of radicals that can chemically link to nearby proteins containing similar moieties, such as tyrosine or tryptophan. Activated agents thus remain bound to tissue sections, while unactivated agents are easily removed through gentle washing steps. This enables activated MAFA to identify areas of elevated MPO activity. To validate the specificity of MAFA for MPO, we conducted experiments using brains harvested from aged wild-type and MPO-knockout (MPO-KO) mice three days post-ischemic stroke. MAFA was employed to detect MPO activity in the ischemic areas of brain sections, and the obtained signal was compared with chlorotyrosine immunostaining—a specific product of MPO activity [38]. In wild-type mouse brains, we observed a significant elevation in MPO activity within the ischemic regions that correlated well with the chlorotyrosine signal. In contrast, virtually no signal was detected by either MAFA or chlorotyrosine immunostaining in the ischemic areas from MPO-KO mouse brains (*p* = 0.0357, Fig. 3B and 3C). This highlights the specificity of MAFA to MPO activity, as its signal matched well with that of chlorotyrosine in both wild-type and MPO-KO mice tissue sections. Given that young naïve mouse brain sections exhibit a mean MAFA signal of 2.3 x 10^9^ (Fig. 4), slightly above that of MPO-KO mice sections (7.5 x 10^8^, considered to be background), we estimated the sensitivity or detection threshold of MAFA to be approximately ~2 x 10^9 in this in vitro/ex vivo setting.

### MPO activity in the brain increases with aging

We next assessed MPO activity in both aged and young adult naïve brains without stroke using MAFA. We found markedly increased MAFA signal in the thalamus region of aged brains compared to that of young adult brains (*p* = 0.0286, Fig. 4A and 4B, Supplementary Fig. 6-1).

Similarly, we found higher Iba1^+^ signals in the thalamus region of aged brains as compared to young adult brains (*p* = 0.0286, Fig. 4A and 4B). MPO ELISA also showed a higher amount of MPO protein in the aged brain compared to that in the young adult brains (*p* = 0.0286, Fig. 4B).

### Recruitment of MPO^+^ cells was altered by aging after an ischemic stroke

**Figure 5.**
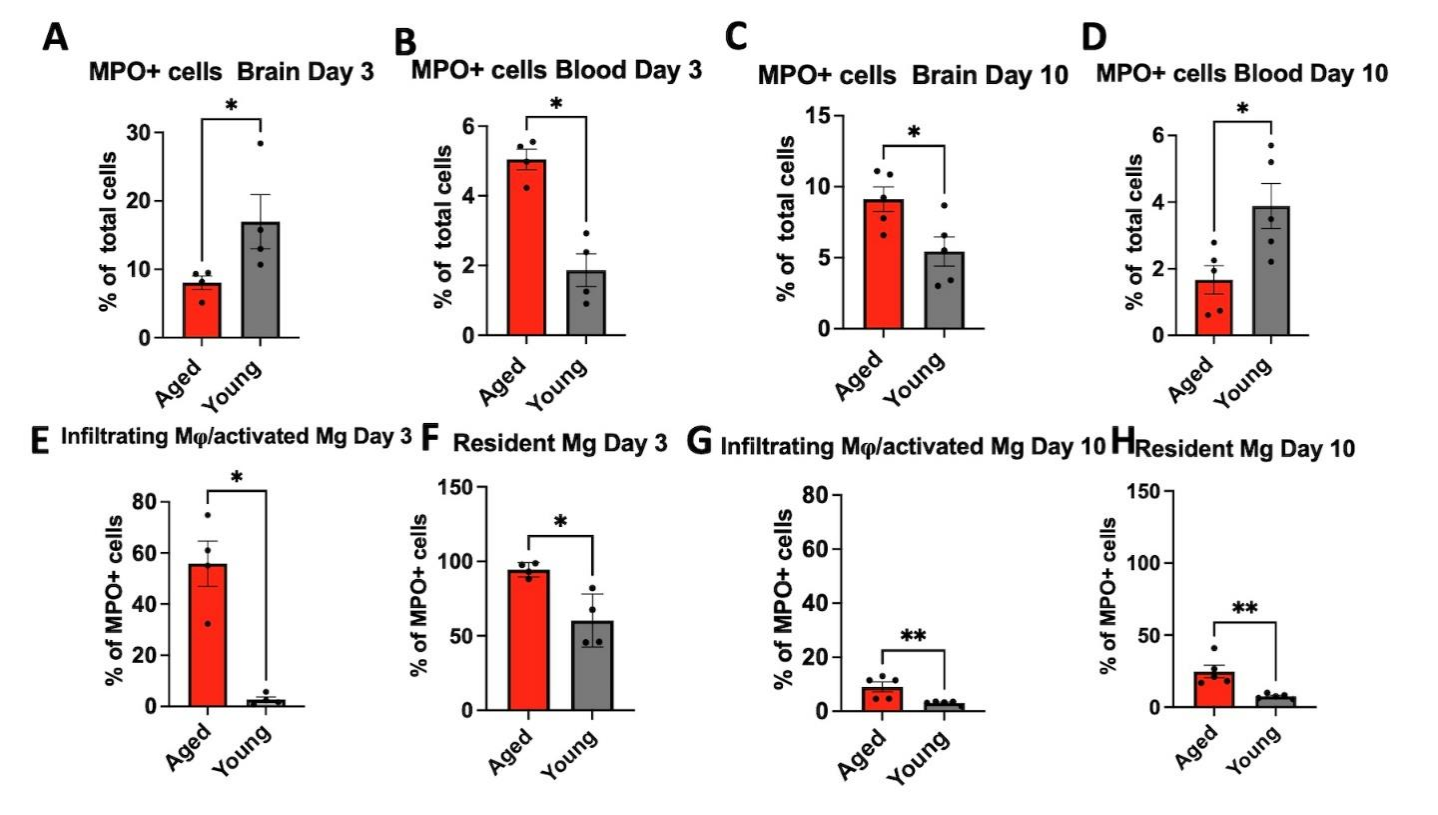
**Aging changes myeloid cell dynamics following ischemic stroke. (A, B)** Analysis of flow cytometry data showed that aged mice have significantly fewer MPO^+^ cells in ischemic brains and more MPO^+^ cells in blood as compared to young adults as compared to young adult brains on day 3 after stroke (n = 4 mice per group). **(C, D)** on day 10^th^ after stroke, aged mice have significantly more MPO^+^ cells in the brain and fewer MPO^+^ cells in the blood as compared to young adults (n = 5 mice per group). **(E, F, G, H)** Notably, the analysis also found a significant increase in MPO^+^ infiltrating M**φ**/activated Mg in the aged brains compared to young adult brains on both day 3 and day 10 post-stroke. Flow cytometry results were quantified as a percentage of total cells and MPO^+^ cells. *, *p* < 0.05, **, *p* < 0.01. Mann-Whitney test was performed to determine the differences between aged and young adults.

We next assessed the effect of aging on the immune response on day 3 (early subacute) and day 10 (late subacute) after ischemic stroke by flow cytometry. We observed a significantly decreased percentage of MPO^+^ cells in the brains of aged mice as compared to that of young adult mice on day 3 after surgery (*p* = 0.0286, Fig. 5A). In contrast, in the blood, a higher percentage of MPO^+^ cells were found in aged mice as compared to those of young adult mice on day 3 after stroke (*p* = 0.0286, Fig. 5B). Interestingly, we observed a significantly increased percentage of MPO^+^ infiltrating macrophages (M**φ**)/activated microglia (Mg) in the brains of aged mice as compared to that of young adult mice on day 3 after surgery (*p* = 0.028, Fig. 5E).

On day 10 post-stroke, a further elevation in the percentage of MPO^+^ cells was evident in the brains of aged mice compared to young mice (*p* = 0.0317, Fig. 5C). Conversely, a decreased percentage of MPO^+^ cells was observed in the blood of aged mice compared to young adult mice on day 10 after stroke (*p* = 0.0317, Fig. 5D). Although both groups experienced a decline in the percentage of MPO^+^ infiltrating M**φ**/activated Mg on day 10 after stroke, the levels remained elevated in the brains of aged mice compared to young adult mice (*p* = 0.0079, Fig. 5E and 5G). Interestingly, the percentage of MPO^+^ resident Mg remained consistently elevated in the aged compared to that of young ischemic brains over time (Fig. 5F, 5H, and Supplementary Fig. 1)

### Aging enhanced the MPO activity after ischemic stroke

**Figure 6.**
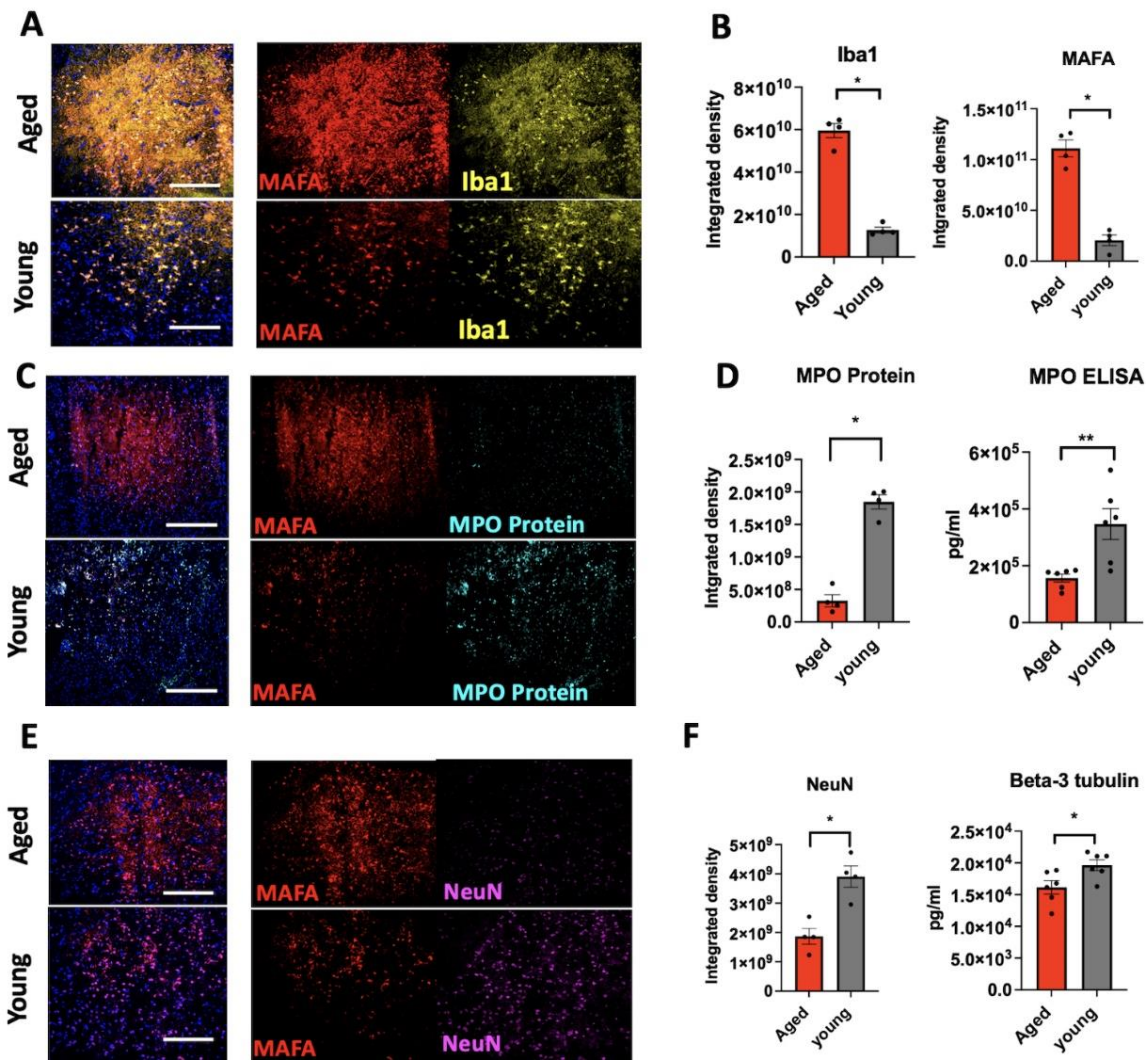
**Immunofluorescent imaging of MPO activity compared to Iba1, MPO protein, and neuronal integrity at the early subacute stage (day 3) after stroke. (A, C, E)** Representative immunofluorescent images depicting MAFA, Iba1, MPO protein and NeuN staining of the infarct area of aged and young adult brains on day 3 after stroke (respectively n = 4 mice per group, the scale bars = 100 µm). **(A, B)** The integrated density of MAFA signal was significantly higher in aged brains as compared to young adults on day 3 after stroke. **(A, B)** The integrated density of Iba1 signal also was higher in aged brains as compared to young adults. **(E, F)** NeuN expression in aged brains is significantly lower on day 3 after stroke as compared to young adult brains. **(D, F)** These results were corroborated with MPO and beta-3 tubulin assays (n = 6 mice per group). *, *p* < 0.05, **, *p* < 0.01). Mann-Whitney test was performed to determine the differences between aged and young adults.

Since we demonstrated that aging affected immune cell recruitment and altered neurological outcomes after stroke, we next investigated how MPO activity changes in aged and young adult brains on days 3 and 10 after stroke using MAFA. We found that aged brains displayed significantly increased MAFA signal on day 3 after stroke compared to that of young adult mice (*p* = 0.0286, Fig. 6A, 6B and Supplementary Fig. 6-2). Correspondingly, we found higher Iba1^+^ signals in aged brains as compared to young adult brains on day 3 after stroke, and colocalized with MAFA signals (*p* = 0.0286, Fig. 6A, 6B and Supplementary Fig. 6-2). Interestingly, double immunofluorescent staining of MPO protein and MAFA showed that aged brains have less MPO protein compared to those of young adult brains on day 3 after stroke (*p* = 0.0286, Fig. 6B and 6D). In addition, the MPO protein foci in the aged brains are much smaller in size compared to those in the young adult brains, revealing that these smaller foci in the aged brain represent granules or lysosomes that have degranulated and secreted MPO. On the other hand, while there were more and larger foci of MPO protein in the young adult brain, there was comparably less MAFA signal, indicating that these MPO^+^ cells have less degree of degranulation. MPO immunostaining results were confirmed by MPO enzyme-linked immunosorbent assay (ELISA) (*p* = 0.0043, Fig. 6D). As expected, the neuronal nuclear protein (NeuN) staining revealed a significant decrease in neuronal integrity in aged brains on day 3 compared to that of younger brains on day 3 (*p* = 0.0286, Fig. 6E, Fig. 6F and Supplementary Fig. 6-2). Similarly, β-3 tubulin ELISA confirmed a similar decrease in the aged brains compared to those from young adult brains on day 3 after stroke (*p* = 0.026, Fig. 6F).

**Figure 7.**
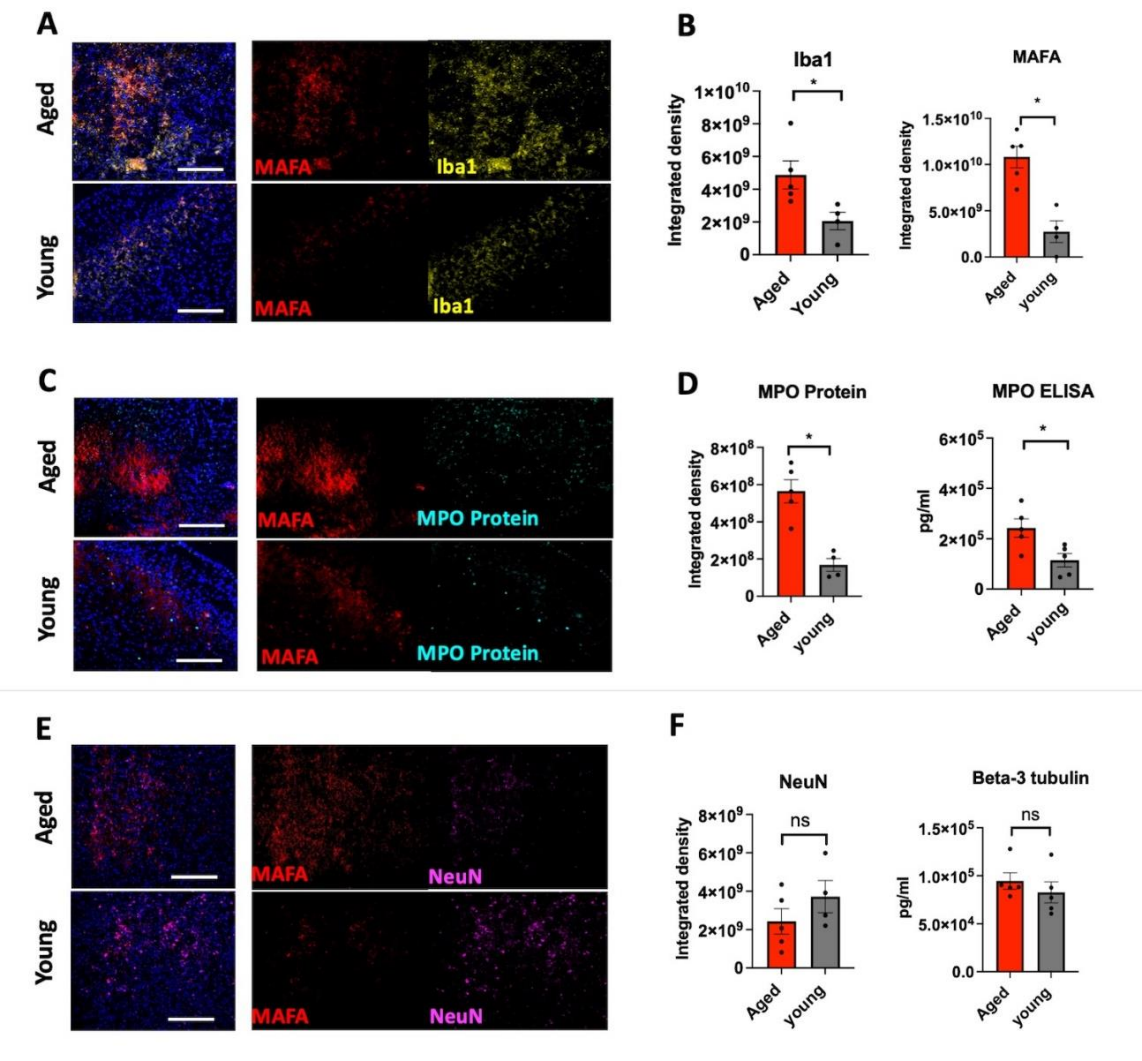
**Immunofluorescent imaging of MPO activity compared to Iba1, MPO protein, and neuronal integrity at the late subacute stage (day 10) after stroke. (A, C, E)** Representative immunofluorescent images showing MAFA, Iba1, MPO protein and NeuN staining of the infarct area of aged and young adult brains on day 10 after stroke (respectively n = 4-5 mice per group, the scale bar = 100 µm). **(A, B)** The integrated density of MAFA was significantly higher in aged brains as compared to young adults on day 10 after stroke. **(A, B)** The integrated density of Iba1 also was higher in aged brains as compared to young adults. **(E, F)** NeuN expression in aged brains trended lower on day 10 after stroke as compared to young adult brains. **(D, F)** These results were corroborated with MPO and beta-3 tubulin assays (n = 5 mice per group). *, *p* < 0.05; ns, not significant. Mann-Whitney test was performed to determine the differences between aged and young adults.

On day 10 after the stroke, while MAFA signal markedly decreased from that of day 3, higher MAFA signal and MPO protein were still detected in the aged brains compared to those of the young adult (Fig. 7A-D and Supplementary Fig. 6-3). Concordant with MPO immunostaining results, the amount of MPO protein was higher in the aged brain as compared to that of the young adult on day 10 after stroke (*p* = 0.0159 and *p* = 0.0317 respectively, Fig. 7B, 7D and Supplementary Fig. 6-3). Similar to MAFA results we found that the Iba1 signal also decreased on day 10 as compared to day 3 in both groups and the Iba1 signal remained significantly higher in aged brains as compared with young adult brains (*p* = 0.0159, Fig. 7A, 6B and Supplementary Fig. 6-3). Notably, only a subset of Iba1^+^ cells showed MAFA signal, indicating that on day 10 not all Iba1^+^ cells are secreting MPO. Similar to day 3 results, we also found discordance between MPO immunostaining and MPO activity (MAFA) imaging, again indicating that MPO immunostaining predominately identifies cells that have not secreted MPO. There were no significant differences between aged and young adult brains in NeuN immunostaining and β-3 tubulin assays on day 10 after stroke (Fig. 7E, 7F, and Supplementary Fig. 6-3). The evolution of MAFA signal over time in different age groups is summarized in Supplementary Fig. 2.

### MPO inhibition decreased MAFA signal and improved behavior outcomes in aged in the early subacute stage following stroke

**Figure 8.**
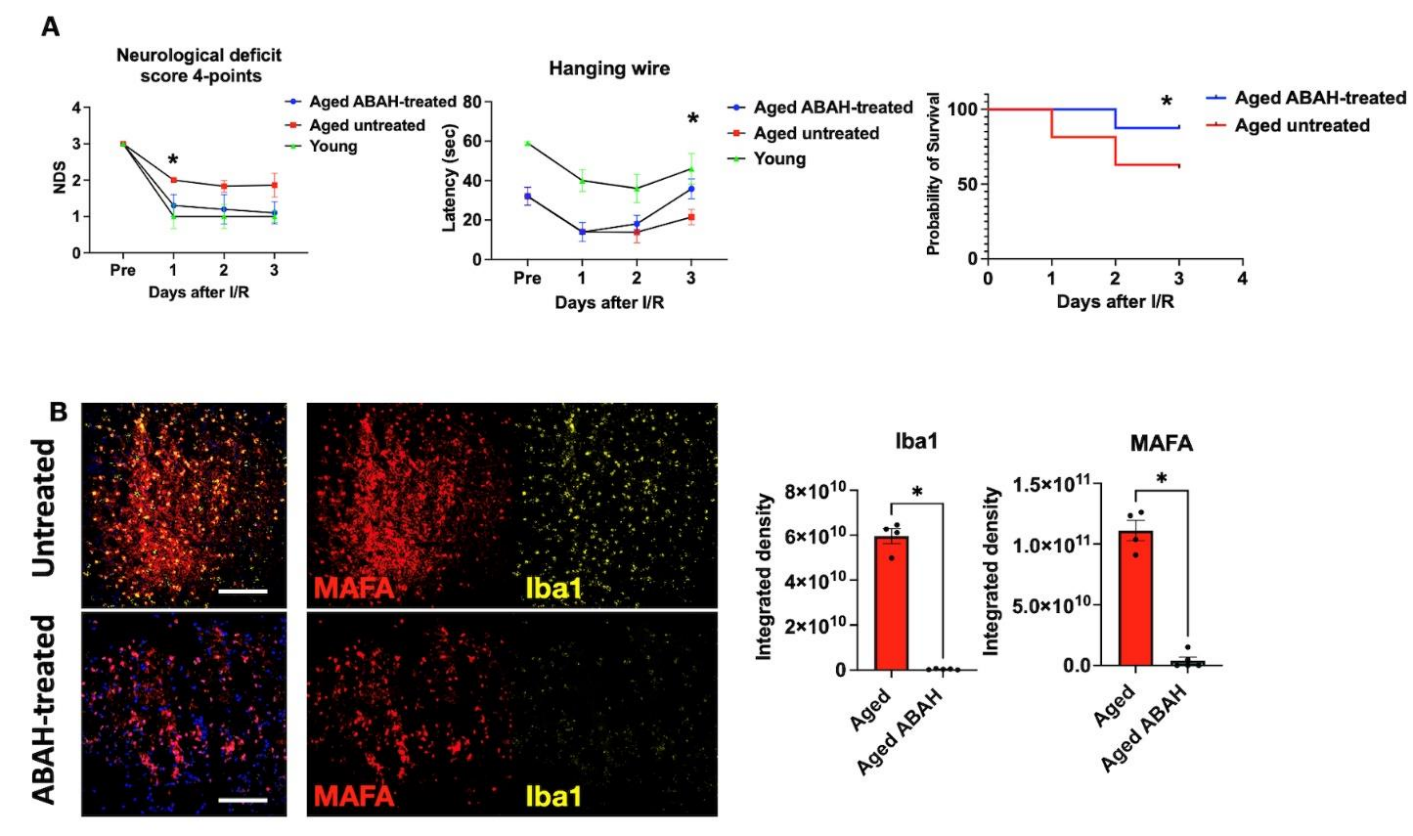
**Neurobehavioral evaluation and survival rate show a beneficial effect of MPO inhibitor on stroke outcome in the aged group**. (A) Behavior and functional tests of aged ABAH-treated and aged untreated and young adult stroke mice during the early subacute stage after stroke (n = 6 ABAH-treated, n = 11 untreated, and n = 6 young adult) and survival (log-rank) curve of aged ABAH-treated (n = 12) and aged untreated mice (n = 55) after stroke. (B) Representative immunofluorescence images showing MAFA and Iba1 signals of infarct areas in ABAH-treated and untreated aged brains on day 3 following stroke (n = 4-5 per group, the scale bar = 100 µm). *, *p* < 0.05. Mann-Whitney test was performed to evaluate the differences of Iba1 and MAFA signals between ABAH-treated and untreated aged stroke mice. Neurological deficit score and hanging wire test between the aged untreated and ABAH-treated groups were analyzed using the unpaired two-tailed t-test.

We next investigated the impact of 4-aminobenzoic acid hydrazide (ABAH), an irreversible and specific MPO inhibitor, on neurobehavior outcomes during the early subacute stage following stroke in aged mice. As expected, MPO tissue imaging on day 3 after stroke revealed decreased MAFA signals in ABAH-treated brains compared to those in untreated brains (*p* = 0.0159, Fig. 8B and Supplementary Fig. 6-4). Similarly, we observed a decrease in Iba1^+^ immunostaining in the ABAH-treated group compared to that of the untreated group (*p* = 0.0159, Fig. 8B and Supplementary Fig. 6-4). Consistent with the decreased inflammatory response, we found that ABAH-treated group exhibited notable improvement in functional outcomes compared to untreated group, approaching the levels of untreated young adult mice after stroke (Fig. 8A). Consequently, we observed a significant improvement in mortality in the ABAH-treated group compared to the control-aged group during the early subacute phase after stroke (*p* = 0.04, Fig. 8A).

## DISCUSSION

While aging is an unmodifiable risk factor for stroke, the adverse changes resulting from aging may be potentially altered. In this study, we developed and utilized the new MAFA agent to track MPO activity on ischemic tissues longitudinally in aged and young adult mice after stroke. Examing how MPO activity in thalamus and cortex regions changed from naïve (pre-stroke), day 3 (post-stroke), and day 10 (post-stroke) brains in aged and young adults, we found that while aging primarily impacts the thalamus and spares the cortex in naïve mice with elevated MPO activity, MPO activity increased in both thalamus and cortex regions after stroke regardless of age (Supplementary Fig. 2). However, there was a much sharper increase in MPO activity in the aged animals in both regions within the first 3 days after ischemia, while the increase in the young adult animals was more modest. Although MPO activity decreased markedly in both aged and young adult mice by day 10 after stroke, it remained elevated in the aged brains compared to the young adult brains. Importantly, this rise in MPO activity after ischemic stroke in the aged group corresponded to the time of highest mortality in this group (within 48 hours). Notably, the administration of ABAH, a specific and irreversible inhibitor of MPO, exhibited remarkable outcomes in mitigating the inflammatory response, reducing MPO activity, enhancing neurological outcomes, and improving the mortality rate in the aged group after stroke. These positive effects played a pivotal role in ameliorating neurobehavior, with noticeable improvement apparent as early as 48 hours post-stroke. These observations suggest that modulating MPO activity during the initial phase has the potential to significantly improve post-stroke outcomes in the elderly population.

We designed MAFA to be an efficient and specific fluorescent probe to detect MPO activity. MAFA is activated by MPO and hydrogen peroxide to form radicals and chemically links to nearby proteins containing tyrosine or tryptophan [38, 39]. In comparison to other methods, such as MABS [40], which serves as a versatile imaging platform but is not specifically designed for fluorescent imaging and requires a secondary reporter imaging probe linked to streptavidin for reporting on MPO activity, MAFA stands out as a self-contained probe that does not require a secondary reporter. This characteristic makes MAFA more convenient for in vitro/ex vivo applications, requiring fewer washing steps. Additionally, in vivo administration requires only a single injection, streamlining the process compared to the two injections needed with MABS. Several other optical imaging agents that may report on MPO have been described but are not specific to MPO activity, including bioluminescent agents luminol [7] and L-012 (designed to detect NADPH oxidase) [41], SNAPF (designed to detect hypochlorous acid and other oxidants but not MPO activity directly) [9]. In this study, MAFA demonstrated the superiority of detecting MPO activity over antibody-based MPO protein immunostaining in identifying the underlying biological process. This is especially crucial in cases where enzymatically active extracellular MPO may not co-localize with MPO protein stored in intracellular granules or lysosomes.

As shown in Fig. 6 and 7, these apparently incongruent results between MPO activity and MPO protein levels are likely because during acute inflammation there is massive degranulation to secrete MPO into the extracellular space. As a result, less MPO is stored intracellularly in the active immune cells to be detected by immunohistochemical techniques as the harsher reagent treatments usually make detecting extracellular targets challenging if not impossible. On the other hand, when MAFA was used to detect MPO activity, it only involved gentle washing steps and the activated MAFA binds to surrounding proteins, which enables detection of extracellular MPO activity. Indeed, we found similar results in a mouse model of cerebritis using an MPO activatable biotinylated sensor [40]. Thus, the aged mice on day 3, with a high MAFA signal but low MPO protein expression in the ischemic brain revealed a high degree of oxidative stress and damaging inflammation. On the other hand, in the young adult mice on day 3, there was a much lower MAFA signal but a high amount of MPO protein, signifying that most of the immune cells were primed but did not release MPO extracellularly to cause damage. These findings were corroborated by NeuN and β-3-tubulin results that showed more damage in the aged mice brains compared with young adult mice brains.

During the late subacute phase (day 10), inflammation has greatly subsided as revealed by the >4-5 folds decrease in MAFA signal on day 10 compared to day 3 for both age groups. However, MPO protein levels were only slightly decreased in the aged brains but markedly decreased in the young adult brains. Together these results revealed that young adults are more resilient toward inflammatory damage after ischemic stroke, with less secreted MPO during the early subacute phase of stroke and a faster resolution as stroke evolved. On the other hand, in the aged mice, ischemic stroke elicited a much larger MPO-mediated response that even during the resolution stage there continued to be MPO^+^ cells in the ischemic area even if they are not actively degranulating.

We observed an increase in MPO activity within the thalamus region in aged brains compared to young adult brains. Previous studies have shown that aging contributes to the heightened vulnerability of both aged mice and elderly humans to microbleeds and thalamic degeneration [42, 43]. Our results provide potential mechanistic insight into the cause of thalamic degeneration. We also found that the increased MPO activity in the thalamus in naïve aged brains colocalized with Iba1^+^ cells, which are likely activated microglia given the lack of injury to recruit peripheral macrophages. After ischemic stroke, more Iba1^+^ cells are recruited to the ischemic sites in both aged and young adult brains, which are likely a mixture of microglia and macrophages. On day 3, Iba1^+^ cells colocalized with MAFA signal, indicating that these cells are predominately MPO-secreting cells and M1-like. By day 10, only a small subset of Iba1^+^ cells colocalized with MAFA signal, most Iba1^+^ cells at this time point are non-MPO-secreting, M2-like, cells performing repair.

Aged mice demonstrated worse initial neurological deficits and exhibited a slower recovery compared to young adult mice after stroke. Nonetheless, aged mice eventually recovered to almost equivalent levels seen in young adult mice by day 10, similar to a previous study [13]. Despite this apparent eventual recovery, aged mice had an increased mortality rate and a decreased survival after stroke, which is also consistent with previous studies [44, 45]. Notably, most deaths in the aged occurred within the first few days after stroke, which is likely related to the markedly elevated MPO activity we observed between days 0 (naïve) to day 3 in the aged group (Supplementary Fig. 3). MPO, as an enzyme known for its high oxidative activity, plays a pivotal role in generating both oxidative and nitrosative stress. When activated by H_2_O_2_, MPO catalyzes the oxidation of substrates (e.g., chloride (Cl̄), bromide (Br̄), nitrite (NO_2_¯), tyrosine (Tyr)), resulting in the production of potent oxidants (hypochlorous acid (HOCl) or hypobromous acid (HOBr)) and free radicals (nitrogen dioxide (?NO_2_) or tyrosyl radical (Tyr?)). These highly reactive species surpass the potency of O_2_-? and H_2_O_2_, leading to cellular injury and increased oxidative stress [1, 2]. Thus, elevated MPO activity serves as an indicator of heightened oxidant production, indicating an excess of oxidative stress. Previous studies suggested that aging involves alterations in membrane fatty acid composition, including a decrease in polyunsaturated fatty acids (PUFAs) and an increase in monounsaturated fatty acids. PUFAs, like arachidonic acid (AA), abundant in the aging brain, are susceptible to free radical attack, and the oxidative depletion of AA levels contributes to cognitive deficits in aged rats [46, 47]. Other studies indicated that protein oxidation, reflected in elevated protein 3-nitro-tyrosine (3-NT) (a downstream effect of MPO activation through the oxidation of nitrite) levels in different regions of the brain of aged animals and white matter of aging monkeys, is implicated in the decline of physiological functioning during aging [48, 49]. Additionally, we found an increased percentage of MPO+ cells in the blood of aged mice following stroke during the early subacute phase, highlighting an age-related amplification of the bone marrow response. This observation aligns with previous studies that established a significant correlation between elevated blood neutrophil counts with more severe neurological deficit scores and higher mortality rates [29]. In addition to increased innate immune response, increased MPO activity has been found to decrease neurogenesis and neuroprotection [18], which likely also contributes to increased mortality in the aged. As MPO activity increases oxidative stress and elevated oxidative stress is associated with aging, aging-related changes and elevated abnormal MPO activity, as we found in the aged mice after stroke, may form a vicious cycle that worsens each other and leads to higher mortality. Thus, MPO activity may have both direct and indirect effects on mortality.

Notably, on the 3rd day post-stroke, a marked decrease was found in both MAFA and Iba1^+^ signals in the aged group after treatment with ABAH, revealing that inhibition of MPO activity resulted in a reduced recruitment of Iba1^+^ cells to the infarcted area. Furthermore, this reduction in MPO activity translated into significant improvement in neurobehavioral performance and mortality rate within the aged group approaching the level seen in young adult stroke mice. Our work has revealed an association between increased MPO activity, particularly in the early subacute phase post-stroke, and neuronal damage in aged mice. Previous findings demonstrated that MPO inhibition, coupled with its impact on oxidative stress and inflammation, fosters an environment that activates crucial endogenous resources, including promoting neurogenesis [18] and neuroprotection [29]. These findings provide evidence linking MPO inhibition with protection against damage and improving repair after stroke. Thus, the aged mice with elevated MPO activity compared to those of young adult mice likely have decreased neuroprotection and neurogenesis capabilities. Future studies investigating the association between MPO activity and neurogenesis and neuroprotection in different age groups will contribute to a more nuanced understanding of the impact of MPO activity across the aging spectrum.

This study has limitations. First, while both male and female animals were included in our study, our study was not powered to analyze potential differences in MPO activity and outcomes between the two sexes within each age group. Sex-specific responses to aging and ischemia have been reported in previous studies, suggesting that females may exhibit a different stroke phenotype than their male counterparts at different ages [44]. We intend to investigate sex differences in the future. Second, we recognize that the 30-minute transient MCAO followed by reperfusion might not mimic all clinical scenarios, although this model may reflect the cases when stroke patients with large vessel occlusion undergo successful thrombectomies or thrombolysis. Future investigations using other models such as thromboembolic stroke could yield additional insights. Because MAFA requires MPO activity, it only works on fresh-frozen tissues as fixation would denature MPO and destroy its activity. Also, as MAFA needs to diffuse into the tissue as well as needing to wash out unactivated MAFA from the tissue, section thickness is a potential limitation if thicker sections are required. However, in our experience, we were still able to perform MAFA imaging in tissue sections up to 1 mm thick.

Elevated systemic levels of MPO have been found to increase mortality in elderly individuals [50]. Our study provides a potential mechanism for this observation in aging humans, bridging a gap in knowledge. Unraveling the relationship between aging, MPO activity, and stroke outcomes could allow more rational design of more effective therapeutic interventions that benefit different age groups after stroke. This understanding becomes crucial in the context of anti-oxidative therapy trials, potentially offering insights to identify drug targets for the elderly and aiding in the identification of suitable patients. Multiple MPO inhibitors are in different stages of clinical development to mitigate oxidative stress and inflammation associated with various diseases [51-53]. However, the translation of MPO inhibition into a viable therapeutic strategy for clinical applications presents its own set of challenges. Critical considerations include determining optimal dosage and managing potential off-target effects. Additionally, the inherent variability among individuals, given that there are different MPO genotypes in humans and likely differential responses to MPO inhibition [54, 55], complicates accurate predictions of responses to MPO inhibition in the absence of a prognostic biomarker to select patients who will most benefit from the therapy. Imaging MPO activity could offer such a biomarker to improve patient selection and track outcomes from MPO inhibition.

## Conclusions

Through longitudinal studies between two different age groups, we demonstrated that elevated MPO activity in aging markedly worsened neurological outcomes after ischemic stroke. We observed that the administration of ABAH, a specific irreversible MPO inhibitor, mitigated the inflammatory response, reduced MPO activity, and improved neurobehavioral scores, contributing to a significant improvement in survival rates by day 3 after stroke in the aged group. These findings underscore the critical role of modulating MPO activity during the initial phase post stroke, emphasizing its potential to significantly enhance post-stroke outcomes in elderly patients. Our findings showed that sustained MPO activity in the aged if left unmitigated, also worsened longer-term outcomes by day 10 and possibly beyond.

## Supplementary Materials

The Supplementary data can be found online at: www.aginganddisease.org/EN/10.14336/AD.2023.1640.
