# Supplementary material for: Aging Intensifies Myeloperoxidase Activity after Ischemic Stroke
Source: Aging Dis. 2024 Aug 30;15(6):2650–64. doi: 10.14336/AD.2023.1640 (PMC11567251; doi:10.14336/AD.2023.1640)
Supplement: Supplementary file 1 — The Supplementary data can be found online at: www.aginganddisease.org/EN/10.14336/AD.2023.1640. [file AD-15-6-2650-s.pdf]

SUPPLEMENTARY DATA

Aging Intensifies Myeloperoxidase Activity after Ischemic Stroke

Negin Jalali Motlagh, Cuihua Wang, Hyung-Hwan Kim, Yonghyun Jun, Daeki Kim, Seeun Lee, John W. Chen

© 2024. Motlagh MJ et al. Published online at http://www.aginganddisease.org/EN/10.14336/AD.2023.1064

SUPPLEMENTARY DATA

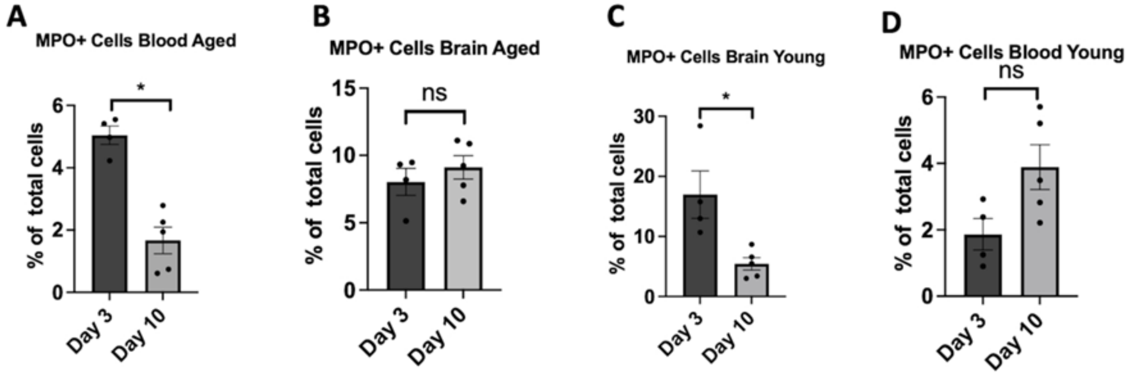

Supplementary Figure 1. The recruitment of MPO^+ cells decreased in young adult brains while there were no changes in aged brains comparing day 10 to day 3. MPO^+ cells in aged blood on day 3 were higher compared to aged blood on day 10 ($p = 0.0073$, Figure A). However, there were no significant differences between day 3 and day 10 in aged brains (Figure B). On the other hand, the percentage of these cells was significantly increased in young adult brains on day 3 compared to day 10 ($p = 0.0159$, Figure C) and there were no significant differences between MPO^+ cells on day 3 and day 10 in young adult blood (Figure D).

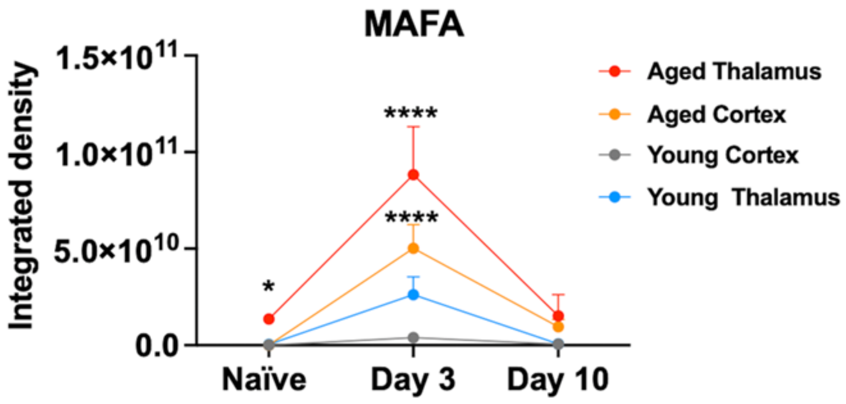

Supplementary Figure 2. Changes in MPO activity in the different ischemic areas over time. Analyses of MAFA imaging in the thalamic and cortical regions of aged and young adult brains revealed a higher MAFA signal in the thalamus and cortex of aged brains irrespective of stroke. These differences in MAFA signals in the naïve thalamus and in Day 3 thalamus and cortex between aged and young adults were statistically significant ($p = 0.0442$ for naïve thalamus, $p < 0.0001$ for thalamus and cortex day 3, n = 4 per group, two-way ANOVA).

score	Body symmetry	Circling behavior	front limb symmentry
0	normal	not present	not present
1	tilting on one side	predomonant oneside turn	slight asymmentry
2	moderate asymmetry	circle to one side not constantly	marked asymmetry
3	promonant asymmetry	circle to one side constantly	prominent asymmetry
4	extreme asymmetry	pivoting or no movenent	no limb movement

Supplementary Figure 3. Neurological deficit score.

© 2024. Motlagh MJ et al. Published online at http://www.aginganddisease.org/EN/10.14336/AD.2023.1064

SUPPLEMENTARY DATA

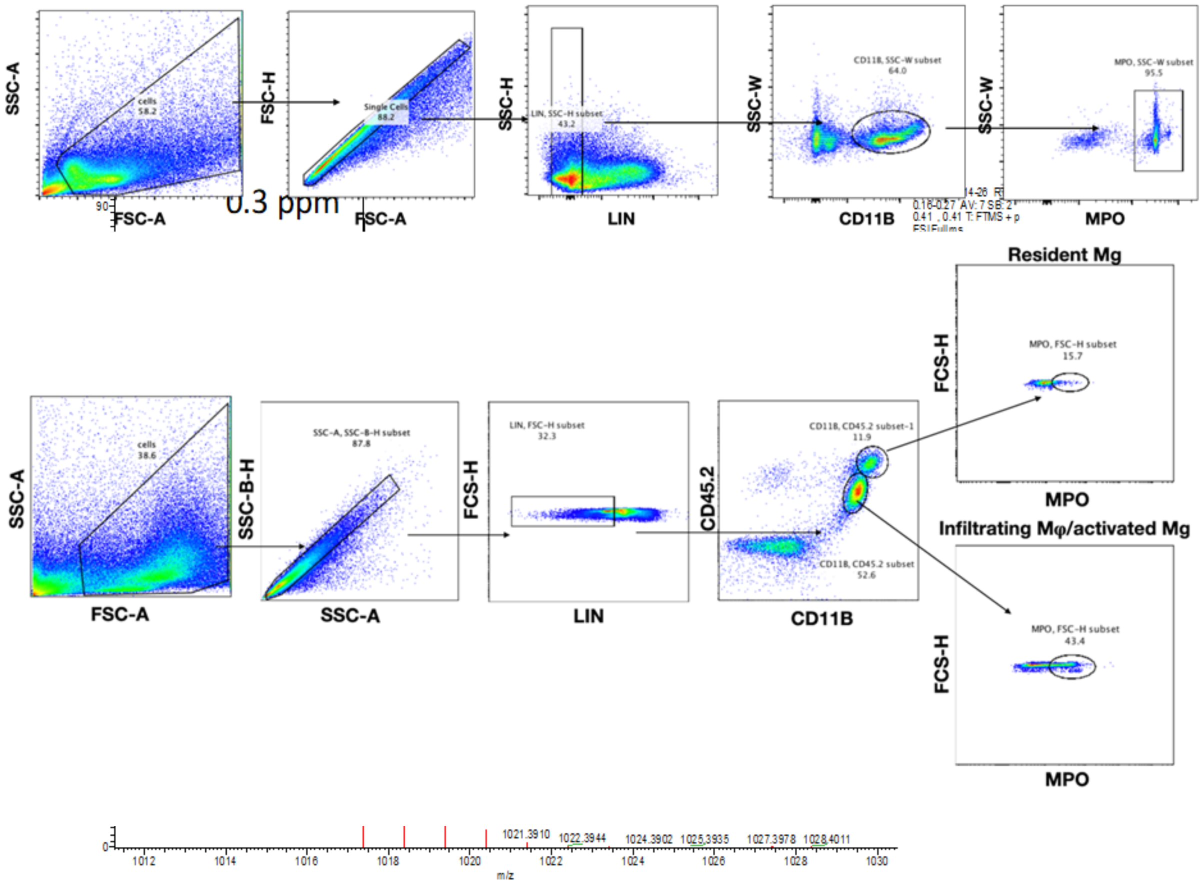

Supplementary Figure 4. Gating scheme for MPO^{+} cells in the brain.

© 2024. Motlagh MJ et al. Published online at http://www.aginganddisease.org/EN/10.14336/AD.2023.1064

SUPPLEMENTARY DATA

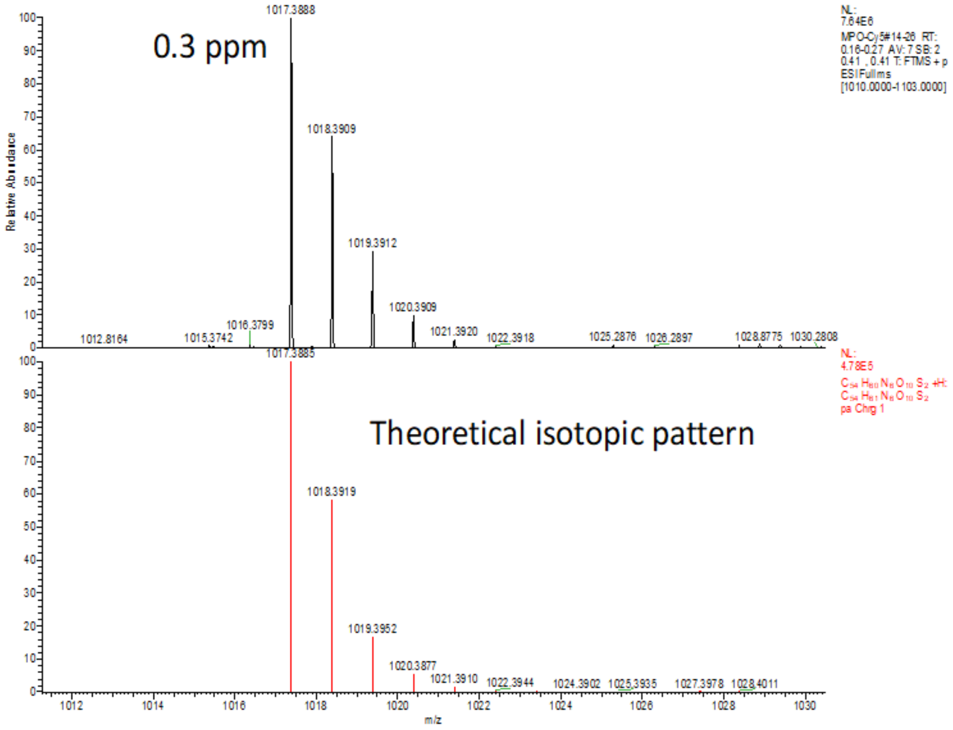

Supplementary Figure 5. High-resolution mass spectrometry (HRMS) of MAFA.

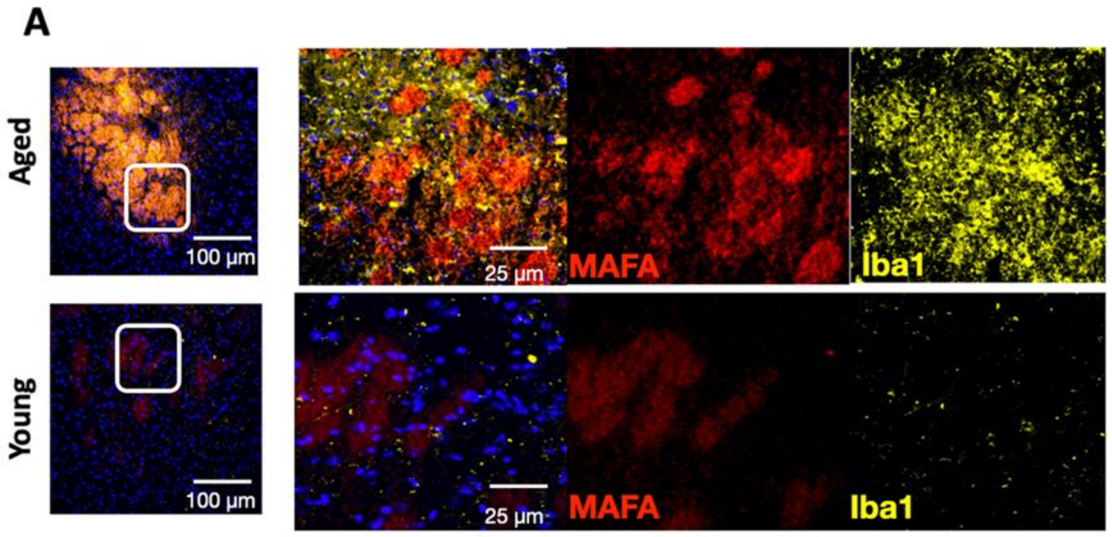

Supplementary Figure 6-1. MAFA signals increased with aging (for Fig. 4).

© 2024. Motlagh MJ et al. Published online at http://www.aginganddisease.org/EN/10.14336/AD.2023.1064

SUPPLEMENTARY DATA

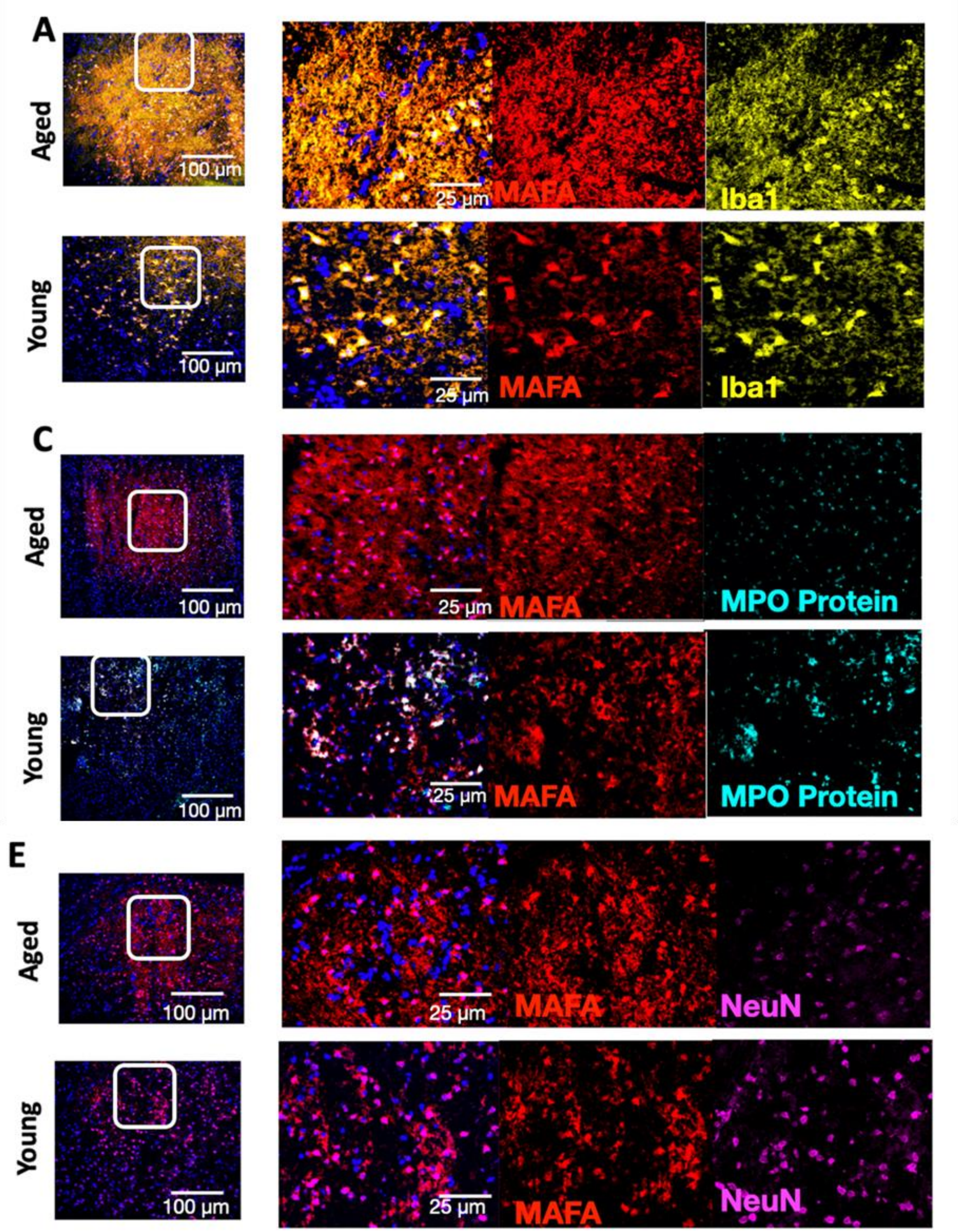

Supplementary Figure 6-2. Immunofluorescent imaging of MPO activity compared to Iba1, MPO protein, and neuronal integrity at the early subacute stage (day 3) after stroke (for Fig. 6).

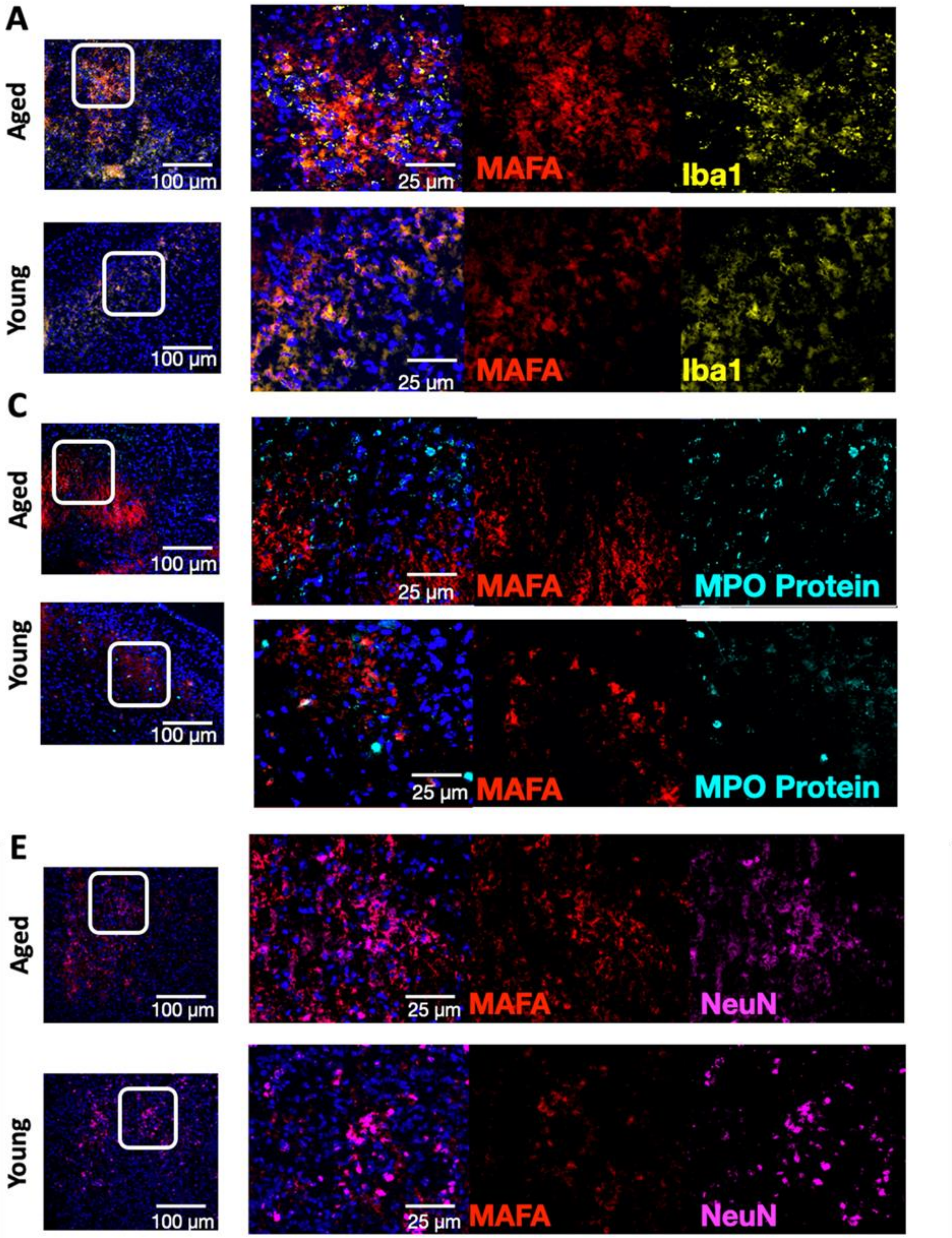

Supplementary Figure 6-3. Immunofluorescent imaging of MPO activity compared to Iba1, MPO protein, and neuronal integrity at the late subacute stage (day 10) after stroke (for Fig. 7).

© 2024. Motlagh MJ et al. Published online at http://www.aginganddisease.org/EN/10.14336/AD.2023.1064

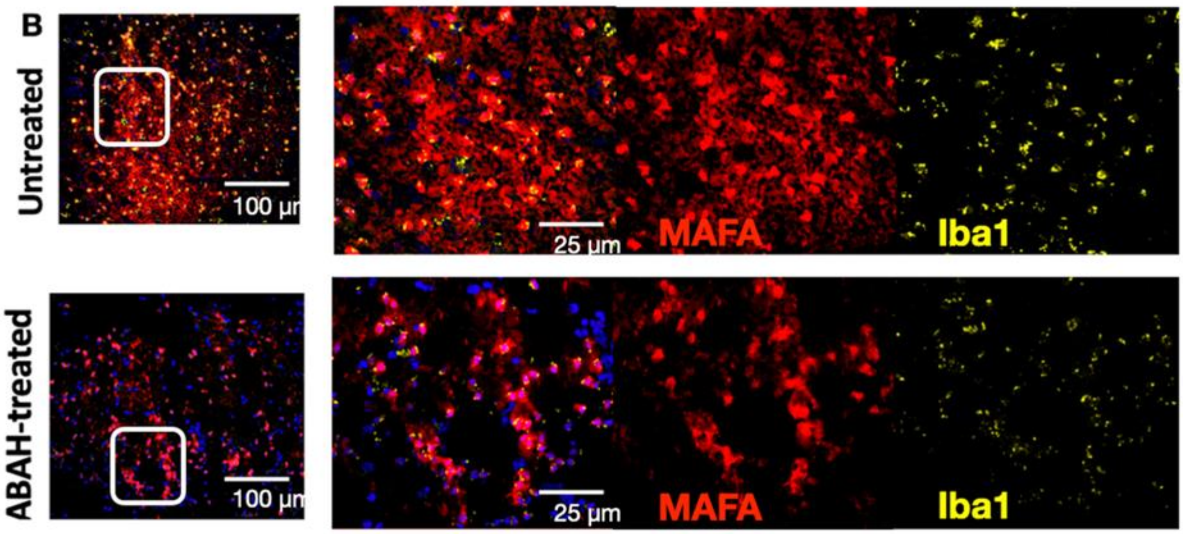

Supplementary Figure 6-4. Neurobehavioral evaluation and survival rate show a beneficial effect of MPO inhibitor on stroke outcome in the aged group (for Fig. 8).
